# Next-generation sequencing approaches for improvement of lactic acid bacteria-fermented plant-based beverages

**DOI:** 10.3934/microbiol.2017.1.8

**Published:** 2017-01-17

**Authors:** Jordyn Bergsveinson, Ilkka Kajala, Barry Ziola

**Affiliations:** 1 Department of Pathology and Laboratory Medicine, College of Medicine, University of Saskatchewan, 2841 Royal University Hospital, 103 Hospital Drive, Saskatoon, SK Canada S7N 0W8; 2 VTT Technical Research Centre of Finland Ltd., PL1000, 02044VTT, Espoo, Finland

**Keywords:** beer, cereal, fermentation, fruit juice, genomics, lactic acid bacteria, legume, metagenomics, soy, transcriptomics, wine

## Abstract

Plant-based beverages and milk alternatives produced from cereals and legumes have grown in popularity in recent years due to a range of consumer concerns over dairy products. These plant-based products can often have undesirable physiochemical properties related to flavour, texture, and nutrient availability and/or deficiencies. Lactic acid bacteria (LAB) fermentation offers potential remediation for many of these issues, and allows consumers to retain their perception of the resultant products as natural and additive-free. Using next-generation sequencing (NGS) or omics approaches to characterize LAB isolates to find those that will improve properties of plant-based beverages is the most direct way to product improvement. Although NGS/omics approaches have been extensively used for selection of LAB for use in the dairy industry, a comparable effort has not occurred for selecting LAB for fermenting plant raw substrates, save those used in producing wine and certain types of beer. Here we review the few and recent applications of NGS/omics to profile and improve LAB fermentation of various plant-based substrates for beverage production. We also identify specific issues in the production of various LAB fermented plant-based beverages that such NGS/omics applications have the power to resolve.

## 1. Introduction

Application of generally-regarded-as-safe or GRAS lactic acid bacteria (LAB) to fermented foods is typically done in pursuit of four goals: flavor modification (including production of alcohol), texture modification, increased nutritional quality (e.g., probiotic LAB strains), and improved food safety and stability (shelf-life). Wide-ranging literature review of these attributes and capacities of LAB in various food and beverage fermentations are performed with reasonable frequency, however, available data is skewed in two respects. First, the bulk of available data is physiological in nature for specific LAB strains, with many singular studies investigating metabolic characteristics of LAB fermentations under narrowly specific conditions. This results in limited understanding of the true microflora and metabolic capacities of LAB associated with these fermentations and stunted ability to identify appropriate starter strains and/or modifications to the fermentation environment in pursuit of improved product. Second, of all fermentations that LAB participate in, dairy fermentations are the most thoroughly studied, due in part to the long involvement of LAB in commercial production of liquid and solid milk products. Consequently, the dairy industry has been subject to the widest application of next-generation sequencing (NGS)/omics to the LAB involved (Table 1), yielding the benefit of well-developed and characterized starter cultures that allow for increased control and optimization of dairy fermentation processes.

Though LAB-fermented dairy products are well studied, milk alternatives produced from raw plant substrates are growing in popularity as increasing numbers of consumers now avoid dairy products for medical or ethical reasons [1],[2]. Such dairy-alternatives can be produced from various cereal or legume sources; however, these substrates can have nutritional or qualitative shortcomings. For example, plant-based beverages can have anti-nutritional issues such as raffinose-family oligosaccharides (raffinose, stachyose, and verbacose), which can cause gastrointestinal discomfort, or phytic acid, which can reduce mineral availability. Most importantly, plant-based proteins may have poor digestibility and often lack some essential amino acids, such as lysine (cereals) or methionine (legumes) [3], and plant substrates may lose some of their water-soluble vitamins during processing [1]. Finally, plant-based milk alternatives can suffer from structural instability caused by a large amount of insoluble compounds like starch, proteins, and dietary fibre [4], to which LAB with their diverse oligosaccharide and protein degrading enzymes can offer remediation. Application of LAB to these products, and other functional beverages (such as those resulting from fruit and vegetable fermentations), thus can often improve upon these quality issues, while maintaining a label-free and *natural* status.

**Table 1.** Next-generation sequencing/omics approaches.

	Approach	Aim	References^1^
Genomics	NGS sequencing of the DNA content of a single organism	To understand the genetic content and coding capacity of a given organism	17–22, 37, 62, 106
Functional genomics	NGS sequencing or other means of interrogation of specific genes of known function in single organisms and/or a mixed microbial community	To understand the presence and distribution of functional genes or known genetic attributes within isolates or a microbial community	82, 85
Metagenomics	NGS sequencing of a mixed microbial community via sequencing a specific genetic marker (i.e., 16S rRNA amplicon) or the total DNA content of a community	To understand the composition (what microbial species are present) and complexity of a microbial community. Provides information as to potential metabolic capacity of the community	28, 39–44, 80, 81, 83, 84
Transcriptomics	NGS sequencing of mRNA transcripts of a single organism growing in a given condition	To understand what genes are expressed and/or required for growth or survival in a given condition	23–25, 36

**^1^** Examples of papers that have utilized the given NGS/omics approach.

In considering LAB in this context, two things must be remembered. First, production of beverages from grains and pulse crops starts with extracting constituents from milled raw material [1], which means that fermentation by LAB will often occur in higher consistency systems (e.g., slurries of raw substrate) than actual beverages. Second, LAB fermentation of milled raw plant materials to develop products with thicker consistency (e.g., yogurt-like non-dairy alternatives) is likely to grow in popularity in coming years. In order to tailor LAB fermentations to achieve desired outcomes, increased application of NGS/omics technologies is required to better understand the variable nature of LAB fermentations of different plant raw substrates from the perspective of what such fermentations can truly accomplish.

Interestingly, although LAB-fermented, dairy-alternative beverages are starting to have an increased omics-research focus, it is LAB involved in plant-based alcoholic beverages such as beer and wine that to date have received the most, albeit still limited, NGS/omics attention. Here we review the specific challenges related to LAB fermentation in production of different types of plant-based beverages - initially beer and wine, and then legumes, cereals, and fruit/vegetables. Additionally, where available, we present recent evidence for using NGS/omics approaches for improving the quality of the products involving LAB fermentation and indicate important future directions for such research.

## 2. Beer

The fermentation of barley, rye, or wheat grain to produce beer is arguably one of the oldest biotechnological processes carried out by humans [5], with perspectives on the role bacteria (and most importantly, LAB) play in brewing shifting over time. For instance, in the production of most beer products available on the market today, LAB are viewed as spoilage organisms, with *Lactobacillus brevis*, *Lactobacillus lindneri*, and *Pediococcus damnosus* being the most commonly encountered bacteria that spoil beer [6]–[10]. Ironically, however, the production of some beer styles, such as Lambic or Flemish sour beers, rely on the free entrance of all manner of microorganisms into the brew (an *open* fermentation) and/or requires purposeful addition of LAB to *sour* the beer by lowering the pH through acid production [11],[12].

Given that LAB have long been viewed as beer-spoilage organisms, extensive literature is available that characterizes the spoilage characteristics of the bacteria involved. This includes detailed description of LAB-induced alterations of the sensorial profile of beer, such as off-flavour formation (diacetyl, acetoin), unwanted acidification, and haze and sediment formation [13]. Unfortunately, to date, the underlying genetic mechanisms of LAB beer spoilage have not been widely resolved, except for the production of rope or slime (exopolysaccharide) by various pediococci species via the *gtf* gene [14], and production of biogenic amines (BAs) by LAB in general [15],[16].

Due to the significance of LAB as spoilage agents of beer, there has been a recent increase in sequenced brewing-related LAB genomes that are publicly available, including the genomes of five *Lactobacillus backii* isolates [17], *L. brevis* BSO 464 [18], *Lactobacillus malefermentans* KCTC 3548 [19], six *P. damnosus* isolates [20],[21], and *Pediococcus claussenii* ATCC BAA-344^T^ [22], as well as upwards of twenty publically available genome projects of isolates associated in some manner to the brewing environment (as of November 1, 2016, via NCBI). Application of transcriptomics to the virulent beer-spoilage organism *L. brevis* BSO 464 (capable of growing in packaged beer with dissolved CO_2_ content/pressure) [23], and to beer-spoiling *P. claussenii* ATCC BAA-344^T^ (can grow in partially degassed beer) [24] has revealed multiple insights into LAB genetic mechanisms in relation to beer-spoilage. And, more recently, both bacteria were analyzed for gene expression when grown in the presence of hops [25]. Notably, these transcriptomic analyses have shown that active metabolism of putrescine and histamine, multiple cellular membrane and wall modifications, and complex transcriptional regulation all occur during active LAB growth in beer. This transcriptomics data, in conjunction with multiple comparative genome studies, also indicates that pentose uptake and utilization of the pentose phosphate pathways is a significant niche adaptation of brewing-related LAB, which is perhaps not surprising given that these sugars are among the major nutrients remaining after yeast-fermentation is finished [23],[25],[26]. Thus, phosphotransferase systems (PTS) genes specific for gluconate and related to the pentose phosphate pathway distinguish beer-spoilage *L. brevis* strains from non-spoiling isolates [23],[26]. In addition to transport proteins with specific action for hop iso-α-acids [9],[27], the plasmid-harbored *fabZ* operon related to fatty acid synthesis can discriminate beer-spoilage *P. damnosus* strains [20]. Further, de novo folate synthesis genes have been found to be common to beer-related pediococci [21] and polygalacturonase, a gene involved in pectin metabolism (i.e., degradation of plant material) distinguishes those isolates capable of growth in beer from isolates not capable of growth in beer [26]. Because of this research, the brewing field has advanced its ability to better identify LAB beer-spoilage strains.

Thus far, only one metagenomic sequencing study has been done in the brewery setting; i.e., on a mixed-culture-fermented beer [28]. However, other techniques such as marker gene detection and LAB-specific terminal restriction fragment length polymorphism have been used to profile the populations of LAB found throughout breweries over time [29]. Similarly, PCR-dependent denaturing gradient gel electrophoresis has been used to detect LAB populations during craft beer production [30]. These studies have revealed greater than anticipated LAB diversity and ubiquity within breweries. This, in turn, has interesting implications for improving process hygiene within the brewery setting and points to the likelihood of parallel LAB diversity in facilities used for production of other LAB-fermented plant beverages.

In an ironic twist, this recent genomics, transcriptomics, and metagenomics data has promise for application in efficiently selecting appropriate starter cultures for the craft fermentations leading to sour beers. As craft beers are emerging popular beverages, their commercial success represents an intriguing opportunity to profile LAB diversity, LAB and yeast co-fermentation, and LAB succession in beer. Further, given the general popularity of beer, opportunities exist to utilize NGS/omics approaches and available NGS/omics data to also improve the functional health (i.e., probiotic) properties of beer [5]. Given that LAB have proven capable of overcoming the harsh growth environment of beer, these bacteria are logical candidates to screen for potential probiotic function and to be involved in both future beer and health research. To date, however, no such follow-up research has been performed.

## 3. Wine

Although malolactic fermentation (MLF) by *Oenococcus oeni* is considered critical for quality wine production, other LAB in the genera *Lactobacillus* and *Pediococcus* can be detected throughout MLF in varying succession patterns [31]. Properties of enological LAB have been well studied, such as the glycosidase enzymes capable of liberating varietal aroma precursors [32],[33] and the esterase enzymes that contribute to fruity aroma and flavour compounds [34]. Indeed, single LAB strains have been profiled for their specific enzymatic activities and the possible impacts on aroma profiles [35]. Despite the knowledge that interactions between yeast and LAB influence the modification of aroma compounds and overall MLF [34],[36], only the genetics underlying *O. oeni* contribution to the taste of wine has been genomically characterized [31]. Indeed, the earliest and most extensive application of genomics technology to plant-based LAB fermentations occurred in the wine industry, with release of the *O. oeni* PSU-1 genome in 2005 [37]. This genomic analysis revealed much about the carbohydrate, nitrate, amino acid, and organic acid (citrate and malate) metabolism of this *O. oeni* isolate. Additionally, the possible stress response of *O. oeni* PSU-1 during growth was determined. Currently, over seventy *O. oeni* genomes and/or genomic projects are publicly available through NCBI [as of November 1, 2016]. At this point, investigation has evolved beyond the genomic study of single *O. oeni* isolates to include the production of a partial proteome reference map for *O. oeni* ATCC BAA-1163 [38], This approach has pointed to important enzymes and proteins coded by the core genome, as well as isolate-specific enzymes such as tributryin esterase (putatively involved in the development of fruity flavor compounds) that require further investigation. Importantly, the writ large potential applications of the wealth of genomic information available for *O. oeni* will begin to be revealed only when coupled with transcriptomics analysis of the bacterium growing under different conditions.

Metagenomic studies have also increased in frequency within the wine industry subsequent to 16S amplicon sequencing being used to determine bacterial diversity in botrytized wine [39]. Similar sequencing methodologies have been employed since to reveal the effect of fermentation and biogeographical influence on both fungal and bacterial communities in grape and wine musts [40], as well as to show how cultivar, vintage and climate condition the microbial populations of wine grapes [41]. Very recently, this type of data has been used to assess the link between wine microbiota, fermentation success and wine properties with the goal of developing new early indicators of quality [42].

Influence of the soil microbiome on grapevine-associated microbiota has also been recently investigated [43], with the finding that the microbiota of leaf and grape correlated with soil carbon. The microbial profile of grape vines was influenced by multiple parameters, with the distribution of microbial taxa again observed to be influenced by biogeographic factors and vineyard management [43], further adding to the body of evidence that grape bacterial communities influence the organoleptic properties of regional wines. As well, metagenomic analysis of grape marc identified *Lactobacillus fabifermentans* as a key organism in the breaking down of residual complex carbohydrates into fermentable sugars during storage-fermentation for the potential production of distilled spirit beverages [44]. This bacterium has one of the largest LAB genomes yet sequenced and possesses a diverse carbohydrate utilization toolbox, with coding for complex gene expression regulation and biofilm formation as important adaptation features related to growth in (fermentation of) grape marc [44].

In addition to identified important carbohydrate, nitrogen, and enzymatic activity, which all contribute important flavour compounds to wine, undesirable metabolic activities of enological LAB have also been identified. For instance, *Oenococcus*, *Lactobacillus, Leuconostoc* and *Pediococcus* all can contribute to the histamine synthesis occurring during wine production [45]. Thus, the danger exists that BA-producing strains might be used as a MLF starter without prior knowledge of their BA-production potential. Consequently, the detection of histidine decarboxylase (or ornithine and/or tyrosine decarboxylase) genes by PCR or DNA probes is now done as a means of wine quality control [46], similar to hop-tolerance genes being screened for as an indication of spoilage-potential for LAB found in beer [47]. Research has also been conducted to identify the genes related to production in wine of ethyl carbamate, a known carcinogen [48], that can be manufactured by LAB in wine during MLF from precursors produced by arginine degradation, such as urea, citrulline, and carbamyl phosphate [48].

Overall, genomic data has clearly aided in the understanding the complex microbial process of wine production. This data, however, has yet to be tapped to streamline and improve the selection of LAB strains with probiotic potential. As with brewing-related LAB, enological LAB must contend with harsh environmental selection pressures such as high sugar content and low pH in grape must, and high ethanol, acidity, SO_2_ content, and limited nutrients in wine [31]; thus, it has been proposed that these LAB may be potential probiotic candidates [49]. This research is within its infancy, with investigation of the in vitro immunomodulatory activities of *O. oeni* and *Pediococcus parvulus* finding that some *O. oeni* strains have measurable immunomodulatory potential, though at a level below *conventional* probiotics [50]. Other results have documented the capacity of 11 wine-related LAB strains to resist lysozyme, gastric juice, and bile to be at levels equal or higher to those observed in the control probiotic strains *Lactobacillus fermentum* CECT5716, *Lactobacillus plantarum* CLC 17, and *Pediococcus pentosaceus* CIAL-86A [51]. As with beer, further application of genetic profiling of candidate probiotic enological LAB can be expected to aid in the development of wine with better functional and even added probiotic properties.

## 4. Legume Beverages

Legumes, particularly soy, offer a popular alternative to traditional dairy products such as milk, yogurt, and cheese; thus, it is not surprising that multiple studies have been conducted analyzing the growth and metabolic behavior of LAB in soy milk [52],[53]. Consequently, the general metabolic and physiological attributes of soy-fermenting LAB have been well established and demonstrated, however, there is limited available research on underlying genetic mechanisms. For instance, many LAB strains possess the metabolic tools to degrade raffinose-family oligosaccharides, a major anti-nutritive issue in legume foods, through α-galactosidase activity of the *mleA* gene [54],[55],[56]. Beyond the identification of this one gene, however, there is limited research on the genetics of raffinooligosaccharide degradation; furthermore, little is known about sequence variability and regulation of *mleA*.

In a similar vein, it is known that the proteolytic specificities of LAB starters for cereal and legume proteins are quite diverse and differ from specificities towards milk proteins [54],[57],[58]. Despite this, little genomic characterization of these proteolytic activities has been performed. This contrasts with the well-studied proteolytic capabilities of LAB and their importance in sensory quality of dairy products [59]. Understanding the genetic mechanisms of proteolytic activity of LAB is important, as this ability can often be exploited to solve quality issues of fermentations. For instance, proteolysis of soy proteins can increase their digestibility or even prevent allergenic problems related to soy [57]. Furthermore, release of lysine from soy protein by some LAB can be used to supplement lysine-deficient cereal foods, thus making nutritionally complete soy-cereal products possible [57]. In this context, genomic, transcriptomic, and metagenomic analysis all have a role to play in selection of specific LAB strains for appropriate proteolytic activity.

Interest in soy beverage production has led to development of strategies to improve the health or nutritional value of product and, in this regard, genetic analysis of specific LAB strains is beginning to demonstrate benefits. For instance, the genetic characterization of riboflavin (vitamin B_2_) biosynthesis pathway of *L. fermentum* has been applied to fortify B_2_ content in soy milk [60], and in soybean matrix by fermentation with a *Lactobacillus reuteri* strain for which a genome sequence was recently published [61],[62]. Other physiological studies that are worthy of genetic exploration include those which demonstrate that specific LAB and *Bifidobacterium* strains improve specific health-related properties of soy, including increasing the antioxidant character [63] and the immuno-modulatory bioactivity of LAB-fermented soy beverage on human intestinal epithelial cells [64].

As an alternative to soy and due to widespread availability, various legumes have been suggested as raw substrates for production of new plant-based beverages and semi-solid foods. For example, fermentation of lupine milk base with *Bifidobacterium animalis* and *L. plantarum* has been shown to obviate the issues posed by both the raffinose family of oligosaccharides and phytic acid, respectively, through degradation of these molecules [65]. Creation of yogurt-like consistencies through LAB fermentation of lupine milk also has been explored [66]. Such important quality improvements of legume substrates by LAB fermentation are paralleled in work where LAB fermentation in sourdough production and effect on phytic acids levels was explored, and phytate degradation by individual strains of *Lactobacillus sanfranciscensis, Lactobacillus pentosus*, and *L. plantarum* was found [67],[68],[69]. Also of note, peanut milk has been established as a suitable growth medium for the LAB yogurt starter cultures *Lactobacillus delbrueckii* subsp. *bulgaricus* and *Streptococcus thermophilus*, with fermentation by these organisms interestingly found to deplete n-hexanal, one of the compounds responsible for the undesirable green and beany flavor of peanut milk [70],[71]. More recently, there has been report of increased antioxidant activity in peanut flour fermented with various *Bifidobacterium* and *Lactobacillus* strains [72]. Although not legume substrates, it should be noted that almond and hazelnut milks also have been demonstrated as potential platforms for fermentation by probiotic LAB strains [73],[74]. Despite these promising studies, and the attractiveness of LAB fermentation to improve the quality of legumes (and nuts) for human consumption, the genetics of LAB in relation to these substrates remains uncharted territory.

## 5. Cereal-based Beverages

Cereal-based, fermented beverages and foodstuffs are traditional and important dietary staples worldwide; see [75] for a comprehensive summary of traditional LAB-fermented cereal foods found in diverse geographic locations, the cereal substrates used, and the LAB involved. Another recent review [2] discusses how LAB can improve cereal-based functional beverages. Despite in-depth and wide-ranging study of metabolic and functional attributes, an unknown degree of variability in the genetics and metabolic capacity of participating LAB remains.

Development of defined LAB starter cultures is of key interest for cereal-based fermentation as it allows for improved product quality, as noted for *Kununzaki* (a non-alcoholic Nigerian beverage) [76],[77], with the use of a defined starter culture resulting in a beverage with improved nutritional quality, aroma, and taste relative to the product produced with spontaneous cultures. However, it has been noted that the replacement of spontaneous or back-slopped starter cultures with defined starter cultures or single strains can result in different sensorial profiles and/or the loss of important flavor characters in cereal-based products [78], specifically so when yeast are not included in the *designer* starter culture [78],[79]. To circumvent this uncertainty, application of metagenomic analysis should be used to initiate the appropriate genomic characterization of natural starters, leading to an understanding of the specific organisms (and their genetic attributes) necessary for successful fermentation such that appropriate designer cultures can be defined.

Recent successful applications in this regard include the metagenomic characterization of the yeast-LAB population for improved production of Chinese rice wine, with the study also demonstrating that product spoilage resulted from the rapid growth of *L. brevis* too early in the fermentation [80]. Metagenomics analysis of the microbial diversity of *xaj-pitha* (also a rice wine) has assisted the development of a suitable fermentation starter culture for this beverage [81], and sequence-based screening approaches have been used to select for rice wine starter cultures with reduced capacity to form BA [82]. Similar analysis of the bacterial diversity and community profile of a traditional fermented Chinese yellow rice wine indicated that LAB comprised a considerably smaller proportion of the population than expected, and that there may be associated safety issues with the microbial community present [83]. Metagenomics have also been used to profile which members of the microbial community of rice wine are responsible for production of volatile compounds in rice wine [84]. Furthermore, functional genomics has been used to analyze fermented pearl millet slurry, a base for several foods in Western Africa, targeting amylolytic, vitamin production, and probiotic survival-related genes to assess the technological potential of the naturally occurring flora [85]. Finally, expression of several amylolytic genes in a *L. plantarum* isolate has been monitored during fermentation of pearl millet slurry [86], providing insight into the required metabolic capacity of LAB during fermentation of this plant substrate.

While metagenomic analysis gains traction within the field of cereal-based beverages, an emerging area of interest is application of this technology to profile the capacity of LAB to produce exopolysaccharides which can be used to improve product texture and stability, as has been studied extensively at the genetic level for sourdough [87],[88]. Recently, Russo et al. [89], demonstrated increased initial viscosity of a fermented oat product by using a genomically characterized potential probiotic *L. plantarum* Lp90 [90]. Other studies have utilized a sequenced strain of *Weisella cibaria* MG1 in production of barley- and soybean-based products to improve both mouth-feel and structural stability [91],[92],[93]. As with legumes, the raw substrate, geographic location, and LAB microflora involved will influence the functional utility of a LAB isolate to a considerable degree. Thus, the genetic characterization of participating LAB is essential for improving specific attributes of each beverage type produced.

## 6. Fruit and Vegetable Juices

Fermented fruit and vegetable juices have favourability and popularity with consumers of all ages and thus make ideal *functional* beverages. Importantly, raw fruit and vegetable substrates contain beneficial nutrients such as minerals, vitamins, dietary fibers, and antioxidants, and do not contain dairy allergens [94],[95]. LAB fermentation of fruit and vegetable substrates can improve levels of desirable flavour compounds and enrich for specific metabolites (e.g., lactic acid, amino acids), while minimizing negative flavour compounds and detoxifying pathogens [96],[97]. However, much remains to be answered about the genetic qualities of LAB that confer appropriate properties to fruit and vegetable-fermented products.

As with cereal-based beverages, fruit and vegetable juices substrates are heterogeneous and diverse, with properties differing across geographic location and fermentation/storage conditions; nonetheless, the general microbial profile of various fruit and vegetable juices have been characterized and reviewed [98]. Many physiological studies are available which have examined production of probiotic juices using a variety of different substrates and LAB strains: orange, pineapple, and cranberry juices with *Lactobacillus casei*, *Lactobacillus rhamnosus* and *Lactobacillus paracasei* [99]; mango juice [100] and noni juice [101] with *L. plantarum*; tomato juice [95] with *Lactobacillus acidophilus*, *L. case*i, *L. delbrueckii*, and *L. plantarum*; red beets with *L. acidophilus* and *L. plantarum* [102]; and cabbage juice with *L. delbrueckii* and *L. plantarum* [103]. In some cases, more detailed analysis has been performed; e.g., the gene-expression of *L. plantarum* in carrot and pineapple juices was studied via micro- and macro-array platforms to understand physiological processes involved in adaptation to the specific juice environment [104],[105] and the importance of carbohydrate and amino acid metabolism regulation, acid tolerance, and pH control was shown. Recent application of genomics and metagenomics have also occurred, with the recent genome announcement for a *Leuconostoc mesenteroides* strain isolated from the Mexican fermented beverage Pulque [106] then allowing for possible probiotic properties associated with this organism to be unravelled [107]. The genome of *Lactobacillus farcimis*, the key organism in the Japanese fermented health beverage *kôso*, has recently been released, underpinning future studies of this bacterium's probiotic and interesting proteolytic capabilities [108]. The same authors have also used metagenomic analysis to profile the microbial community throughout *kôso* fermentation [109]. This analysis revealed the community to be markedly diverse and notably different from the community profile revealed using traditional culture methods, importantly showing that difficult-to-culture bacterial species participate in this fermentation [109].

While metagenomics has begun to reveal specific characteristics of LAB fermentation, it was noted previously that the microflora of juice (and other products) establish in the following sequence as fermentation progresses and growth inhibitory qualities increase in strength: non-fermentative psychrotrophic Gram-negative bacteria → fermentative Gram-negative bacteria → LAB → yeasts → filamentous fungi [110]. Thus, one area that has been explored physiologically is how LAB from fruit might be exploited for production of anti-fungal compounds, as has been shown for *L. fermentum*, *L. plantarum*, and spoilage fungi in tomato fruit [111], and for other LAB strains in plumb, pear, and grape models [112]. A detailed description of the anti-fungal compounds produced has not been made and, most importantly, their production by LAB has not been genetically characterized. The result is that screening of LAB for production of antifungal compounds is still reliant on laborious large-scale growth and physiological studies.

## 7. Conclusions

Plant-based beverages produced via LAB fermentation can consist of a nearly infinite number of compositions and associated issues. This means the LAB involved must possess specific capacities to confer desirable (improved) properties to these beverages. Selecting appropriate LAB and/or developing *designer* LAB starter cultures for such fermentations can only be done with detailed knowledge of attributes individual LAB possess. In this context, the identification and basic characterization of the native microbial flora involved in spontaneously fermented foods and then determining their individual isolate metabolic capacity through traditional microbiology research is a useful starting point. Available nucleotide sequencing technologies then have the power to rapidly reveal important genetic detail for the LAB involved in these fermentations; to date, however, there has been limited output of such research. Nonetheless, in the few instances where NGS/omics have been applied, the data obtained clearly reveal the limitations of traditional metabolic and physiological studies to fully describe complexity of LAB metabolic processes taking place during the fermentations used to produce plant-based beverages. This is clearly exemplified within the fields of brewing and oenology, wherein application of genomic and transcriptomic techniques has demonstrated benefit for increasing the ability to not only rapidly screen for spoilage LAB organisms, but importantly to also provide genetic information that can be used for product innovation through LAB fermentation. NGS/omics data obtained for brewing and enological LAB thus has the potential to not only assist in increasing the functional attributes of beer and wine, but also the expanding assortment of plant-based beverages in general. Through increased application of currently available genomics, transcriptomics, and metagenomics technologies to the LAB used in the production of diverse plant-based beverages, better product outcomes for LAB fermentation can be achieved, regardless of the raw plant substrate involved.

## 

## References

[1] Mäkinen O, Wanhalinna V, Zannini E (2016). Foods for special dietary needs: Non-dairy plant-based milk substitutes and fermented dairy-type products. Crit Rev Food Sci Nutr.

[2] Peyer LC, Zannini E, Arendt EK (2016). Lactic acid bacteria as sensory biomodulators for fermented cereal-based beverages. Trends Food Sci Technol.

[3] Friedman M (1996). Nutritional value of proteins from different food sources: A review. J Agric Food Chem.

[4] Durand A, Franks GV, Hosken RW (2003). Particle sizes and stability of UHT bovine, cereal and grain milks. Food Hydrocol.

[5] Preedy VR, Preedy VR (2011). Beer in health and disease production.

[6] Back W, Back W (2005). Colour atlas and handbook of beverage biology.

[7] Menz G, Andrighetto C, Lombardi A (2010). Isolation, identification, and characterisation of beer-spoilage lactic acid bacteria from microbrewed beer from Victoria, Australia. J Inst Brew.

[8] Priest FG, Priest FG, Campbell I (2003). Gram-positive brewery bacteria. Brewing Microbiology.

[9] Suzuki K, Asano S, Iijima K (2008). Sake and beer spoilage lactic acid bacteria—A review. J Inst Brew.

[10] Thelen K, Beimfohr C, Snaidr J (2006). Evaluation study of the frequency of different beer-spoiling bacteria using the VIT analysis. Tech Q Master Brew Assoc Am.

[11] Spitaels F, Wieme AD, Janssens M (2014). The microbial diversity of traditional spontaneously fermented lambic beer. PLoS One.

[12] Tonsmeire M, Tonsmeire M (2014). Sour beers: A primer. American sour beers: innovative techniques for mixed fermentations.

[13] Bokulich NA, Bamforth CW (2013). The microbiology of malting and brewing. Microbiol Mol Biol Rev.

[14] Pittet V, Morrow K, Ziola B (2011). Ethanol tolerance of lactic acid bacteria, including relevance of the exopolysaccharide gene, *gtf*. J Am Soc Brew Chem.

[15] Kalač P, Šavel J, Křížrek M (2002). Biogenic amine formation in bottled beer. Food Chem.

[16] Kalač P, Křížrek M (2003). A review of biogenic amines and polyamines in beer. J Inst Brewing.

[17] Geissler AJ, Behr J, Vogel RF (2016). Multiple genome sequences of the important beer-spoiling species *Lactobacillus backii*. Genom Announ.

[18] Bergsveinson J, Friesen V, Ewen E (2015). Genome sequence of rapid beer-spoiling isolate *Lactobacillus brevis* BSO 464. Genom Announ.

[19] Kim DW, Choi SH, Kang A (2011). Draft genome sequence of *Lactobacillus malefermentans* KCTC 3458. J Bacteriol.

[20] Behr J, Geissler AJ, Schmid J (2016). The identification of novel diagnostic marker genes for the detection of beer-spoiling *Pediococcus damnosus* strains using the BlAst diagnostic gene finder. PLoS One.

[21] Snauwaert I, Stragier P, De Vuyst L (2015). Comparative genome analysis of *Pediococcus damnosus* LMG 28219, a strain well adapted to the beer environment. BMC Genom.

[22] Pittet V, Abegunde T, Marfleet T (2012). Complete genome sequence of the beer spoilage organism *Pediococcus claussenii* ATCC BAA-344^T^. J Bacteriol.

[23] Bergsveinson J, Friesen V, Ziola B (2016). Transcriptome analysis of beer-spoiling *Lactobacillus brevis* BSO 464 in degassed and gassed beer. Int J Food Micro.

[24] Pittet V, Phister TG, Ziola B (2013). Transcriptome sequence and plasmid copy number analysis of the brewery isolate *Pediococcus claussenii* ATCC BAA-344^T^ during growth in beer. PLoS One.

[25] Bergsveinson J, Ewen E, Friesen V (2016). Transcriptional activity and role of plasmids of *Lactobacillus brevis* BSO 464 and *Pediococcus claussenii* ATCC BAA-344^T^ during growth in the presence of hops. AIMS Microbiol.

[26] Behr J, Geissler AJ, Preissler P (2015). Identification of ecotype-specific marker genes for categorization of beer-spoiling *Lactobacillus brevis*. Food Microbiol.

[27] Sami M, Yamashita H, Hirono T (1997). Hop-resistant *Lactobacillus brevis* contains a novel plasmid harboring a multidrug resistance-like gene. J Ferment Bioeng.

[28] Snauwaert I, Roels SP, Van Nieuwerburg F (2016). Microbial diversity and metabolite composition of Belgian red-brown acidic ales. Int J Food Microbiol.

[29] Bokulich NA, Bergsveinson J, Ziola B (2015). Mapping microbial ecosystems and spoilage-gene flow in breweries highlights patters of contamination and resistance. eLife.

[30] Garofalo C, Osimani A, Milanović V (2015). The occurrence of beer spoilage lactic acid bacteria in craft beer production. J Food Sci.

[31] Bartowsky EJ (2009). Bacterial spoilage of wine and approaches to minimize it. Lett Appl Microbiol.

[32] Gagné S, Lucas PM, Perello MC (2011). Variety and variability of glycosidase activities in an *Oenococcus oeni* strain collection tested with synthetic and natural substrates. J Appl Microbiol.

[33] Milchlmayr H, Nauer S, Brandes W (2012). Release of wine monoterpenes from natural precursors by glycosidases from *Oenococcus oeni*. Food Chem.

[34] Antalick G, Perello MC, de Revel G (2012). Characterization of fruity aroma modifications in red wines during malolactic fermentation. J Agric Food Chem.

[35] Lerm E, Engelbrecht L, du Toit M (2011). Selection and characterisation of *Oenococcus oeni* and *Lactobacillus plantarum* South African wine isolates for use as malolactic fermentation starter cultures. South Afr J Enol Vit.

[36] Rossouw D, du Toit M, Bauer FF (2012). The impact of co-inoculation with *Oenococcus oeni* on the transcriptome of *Saccharomyces cerevisiae* and on the flavour active metabolite profiles during fermentation in synthetic must. Food Microbiol.

[37] Mills DA, Rawsthorne H, Parker C (2005). Genomic analysis of *Oenococcus oeni* PSU-1 and its relevance to winemaking. FEMS Microbiol Rev.

[38] Mohedano ML, Russo P, de los Rios V (2014). A partial proteome reference map of the wine lactic acid bacterium *Oenococcus oeni* ATCC BAA-1163. Open Biol.

[39] Bokulich NA, Joseph CML, Allen G (2012). Next-generation sequencing reveals significant bacterial diversity of botrytized wine. PLoS One.

[40] Pinto C, Pinho D, Cardoso R (2015). Wine fermentation microbiome: a landscape from different Portuguese wine appellations. Front Microbiol.

[41] Bokulich NA, Thorngate JH, Richardson PM (2014). Microbial biogeography of wine grapes is conditioned by cultivar, vintage, and climate. PNAS.

[42] Bokulich NA, Collins TS, Masarweh C (2016). Associations among wine grape microbiome, metabolome, and fermentation behavior suggest microbial constribution to regional wine characteristics. mBio.

[43] Zarraonaindia I, Owens SM, Weisenhorn P (2015). The soil microbiome influences grapevine-associated microbiota. mBio.

[44] Campanaro S, Treu L, Vendramin V (2014). Metagenomic analysis of the microbial community in fermented grape marc reveals that *Lactobacillus fabifermentans* is one of the dominant species: insights into its genome structure. Appl Microbiol Biotechnol.

[45] de Nadra MCM, Mèndez-Vilas A (2007). Nitrogen metabolism in lactic acid bacteria from fruits: a review. Comm Curr Edu Top Trend Appl Microbiol.

[46] Lonvaud-Funel A (2001). Biogenic amines in wines: role of lactic acid bacteria. FEMS Microbiol Lett.

[47] Haakensen M, Schubert A, Ziola B (2008). Multiplex PCR for putative *Lactobacillus* and *Pediococcus* beer-spoilage genes and ability of gene presence to predict growth in beer. J Am Soc Brew Chem.

[48] Araque I, Gil J, Carreté R (2009). Detection of *arc* genes related with ethyl carbamate precursors in wine lactic acid bacteria. J Agric Food Chem.

[49] Spano G, Massa S (2006). Environmental stress response in wine lactic acid bacteria: beyond *Bacillus subtilus*. Crit Rev Microbiol.

[50] Foligne J, Dewulf J, Breton O (2010). Probiotic properties of non-conventional lactic acid bacteria immunomodulation by *Oenococcus oeni*. Int J Food Microbiol.

[51] García-Ruiz A, González de Llano D, Esteban-Fernández (2014). Assessment of probiotic properties in lactic acid bacteria isolated from wine. Food Microbiol.

[52] Mital BK, Steinkraus KH (1979). Fermentation of soy milk by lactic acid bacteria: A review. J Food Protect.

[53] Wang YC, Yu RC, Chou CC (2002). Growth and survival of bifidobacteria and lactic acid bacteria during the fermentation and storage of cultured soymilk drinks. Food Microbiol.

[54] Donkor ON, Henriksson A, Vasiljevic T (2007). α-galactosidase and proteolytic activities of selected probiotic and dairy cultures in fermented soymilk. Food Chem.

[55] LeBlanc JG, Garro MS, Silvestroni A (2004). Reduction of α-galactooligosaccharides in soyamilk by *Lactobacillus fermentum* CRL 722: in vitro and in vivo evaluation of fermented soyamilk. J Appl Microbiol.

[56] Silvestroni A, Connes C, Sesma F (2012). Characterization of the *melA* locus for α-galactosidase in *Lactobacillus plantarum*. Appl Environ Microbiol.

[57] Aguirre L, Herbert EM, Garro MS (2014). Proteolytic activity of *Lactobacillus* strains on soybean proteins. Food Sci Technol.

[58] Pescuma M, Turbay MBE, Mozzi F (2013). Diversity in proteinase specificity of thermophilic lactobacilli as revealed by hydrolysis of dairy and vegetable proteins. Appl Microbiol Biotechnl.

[59] Savijoki K, Ingmer H, Varmanen P (2006). Proteolytic systems of lactic acid bacteria. Appl Microbiol Biotechnol.

[60] Juarez del Valle M, Laiño JE, Savoy de Giori G (2014). Riboflavin producing lactic acid bacteria as a biotechnological strategy to obtain bio-enriched soymilk. Food Res Int.

[61] Molina V, Médici M, de Valdez GF (2012). Soybean-based functional food with vitamin B 12-producing lactic acid bacteria. J Funct Foods.

[62] Torres AC, Suárez NE, Font G (2016). Draft genome sequence of *Lactobacillus reuteri strain* CRL 1098, an interesting candidate for functional food development. Genom Announ.

[63] Wang YC, Yu RC, Chou CC (2006). Antioxidative activities of soymilk fermented with lactic acid bacteria and bifidiobacterium. Food Microbiol.

[64] Wagar LE, Champagne CP, Buckley ND (2009). Immunomodulatory properties of fermented soy and dairy milks prepared with lactic acid bacteria. J Food Sci.

[65] Fritsch C, Vogel RF, Toelstede S (2015). Fermentation performance of lactic acid bacteria in different lupin substrates—influence and degradation ability of antinutritives and secondary metabolites. J Appl Microbiol.

[66] Hickisch A, Beer R, Vogel RF (2016). Influence of lupin-based milk alternative heat treatment and exopolysaccharide-producing lactic acid bacteria on the physical characteristics of lupin-based yogurt alternatives. Food Res Int.

[67] De Angelis M, Gallo G, Corbo MR (2003). Phytase activity in sourdough lactic acid bacteria: purification and characterization of a phytase from *Lactobacillus sanfranciscensis* CB1. Int J Food Microbiol.

[68] Palacios MC, Haros M, Rosell CM (2005). Characterization of an acid phosphatase from *Lactobacillus pentosus*: regulation and biochemical properties. J Appl Microbiol.

[69] Reale A, Mannina L, Tremonte P (2004). Phytate degradation by lactic acid bacteria and yeasts during the wholemeal dough fermentation: a 31P NMR study. J Agric Food Chem.

[70] Lee C, Beuchat LR (1991). Changes in chemical composition and sensory qualities of peanut milk fermented with lactic acid bacteria. Int J Food Microbiol.

[71] Schaffner DW, Beuchat LR (1986). Fermentation of aqueous plant seed extracts by lactic acid bacteria. Appl Environ Microbiol.

[72] Wang NF, Yan Z, Li CY (2011). Antioxidant activity of peanut flour fermented with lactic acid bacteria. J Food Biochem.

[73] Bernat N, Cháfer M, Chiralt A (2014). Hazelnut milk fermentation using probiotic *Lactobacillus rhamnosus* GG and inulin. Int J Food Sci Technol.

[74] Bernat N, Cháfer M, Chiralt A (2015). Almond milk fermented with different potentially probiotic bacteria improves iron uptake by intestinal epithelial (Caco-2) cells. Int J Food Stud.

[75] Blandino A, Al-Aseeri ME, Pandiella SS (2003). Cereal-based fermented foods and beverages. Food Res Int.

[76] Agarry O, Nkama O, Akoma O (2010). Production of *Kununzaki* (a Nigerian fermented cereal beverage) using starter culture. Int Res J Microbiol.

[77] Vieira-Dalodé G (2008). Use of starter cultures of lactic acid bacteria and yeasts as inoculum enrichment for the production of *gowé*, a sour beverage from Benin. Afr J Microbiol Res.

[78] Onyago C, Bley T, Raddatz H (2004). Flavour compounds in backslop fermented *uji* (an East African sour porridge). Euro Food Res Technol.

[79] Muyanja CMBK, Narhvus JA, Langsrud T (2012). Organic acids and volatile organic compounds produced during traditional and starter culture fermentation of *Bushera*, a Ugandan fermented cereal beverage. Food Biotechnol.

[80] Hong X, Chen J, Liu L (2016). Metagenomic sequencing reveals the relationship between microbiota composition and quality of Chinese Rice Wine. Sci Rep.

[81] Sankar Bora S, Keot J, Das S (2016). Metagenomics analysis of microbial communities associated with a traditional rice wine starter culture (*Xaj-pitha*) of Assam, India. 3 Biotech.

[82] Liu SP, Yu JX, Wei XL (2016). Sequencing-based screening of functional microorganism to decrease the formation of biogenic amines in Chinese rice wine. Food Cont.

[83] Fang RS, Dong YC, Chen F (2015). Bacterial diversity analysis during the fermentation processing of traditional Chinese yellow rice wine revealed by 16S rDNA 454 pyrosequencing. J Food Sci.

[84] Liu SP, Mao J, Liu YY (2015). Bacterial succession and the dynamics of volatile compounds during the fermentation of Chinese rice wine from Shaoxing region. World J Microbiol Biotechnol.

[85] Turpin W, Humblot C, Guyot JP (2011). Genetic screening of functional properties of lactic acid bacteria in a fermented pearl millet slurry and in the metagenome of fermented starchy foods. Appl Environ Microbiol.

[86] Humblot C, Turpin W, Chevalier F (2014). Determination of expression and activity of genes involved in starch metabolism in *Lactobacillus plantarum* A6 during fermentation of a cereal-based gruel. Int J Food Microbiol.

[87] Leemhuis H, Pijning T, Dobruchowska JM (2013). Glucansucrases: Three-dimensional structures, reactions, mechanisms, α-glucan analysis and their implications in biotechnology and food applications. J Biotechnol.

[88] Tieking M, Gänzle MG (2005). Exopolysaccharides from cereal-associated lactobacilli. Trends Food Sci Technol.

[89] Russo P, de Chiara MLV, Capozzi V (2016). *Lactobacillus plantarum* strains for multifunctional oat-based foods. LWT-Food Sci Technol.

[90] Lamontanara A, Caggianiello G, Orrù L (2015). Draft genome sequence of *Lactobacillus plantarum* Lp90 isolated from wine. Genom Announ.

[91] Lynch KM, Lucid A, Arendt EK (2015). Genomics of *Weissella cibaria* with an examination of its metabolic traits. Microbiol.

[92] Zannini E, Mauch A, Galle S (2013). Barley malt wort fermentation by exopolysaccharide-forming *Weissella cibaria* MG1 for the production of a novel beverage. J Appl Microbiol.

[93] Zannni E, Zannin E (2015). Chapter 5: Impact of exopolysaccharide-producing *Weissella cibaria* MG1 on the properties of soy yoghurt. Functional application of lactic acid bacteria exopolysaccharide in complex food systems.

[94] Luckow T, Delahunty C (2004). Which juice is “healthier”? A consumer study of probiotic non-dairy juice drinks. Food Qual Pref.

[95] Yoon KY, Woodams EE, Hang YD (2004). Probiotification of tomato juice by lactic acid bacteria. J Microbiol.

[96] Maki M, Salminen S, Von Wright A, Ouwehand A (2004). Lactic acid bacteria in vegetables fermentation. Lactic Acid Bacteria Microbiological and Functional Aspects.

[97] Urbonaviciene D, Viskelis P, Bartkiene E, Ekinci D (2015). The use of lactic acid bacteria in the fermentation of fruits and vegetables—technological and functional properties. Biotechnology.

[98] Di Cagno R, Filannino P, Gobetti M, Mozzi F, Raya RR, Vignolo GM (2015). Chapter 14: Vegetable and fruit fermentation by lactic acid bacteria. Biotechnology of Lactic Acid Bacteria: Novel Applications.

[99] Sheehan VM, Ross P, Fitzgerald GF (2007). Assessing the acid tolerance and the technological robustness of probiotic cultures for fortification in fruit juices. Innov Food Sci Emerg Technol.

[100] Reddy LV, Min JH, Wee YJ (2015). Production of probiotic mango juice by fermentation of lactic acid bacteria. Microbiol Biotechnol Lett.

[101] Wang CY, Ng CC, Su H (2009). Probiotic potential of noni juice fermented with lactic acid bacteria and bifidobacteria. Int J Food Sci Nutr.

[102] Yoon KY, Woodams EE, Hang YD (2005). Fermentation of beet juice by beneficial lactic acid bacteria. Lebensm-Wiss Technol.

[103] Yoon KY, Woodams EE, Hang YD (2006). Production of probiotic cabbage juice by lactic acid bacteria. Biores Techol.

[104] Filannino P, Di Cagno R, Crecchio C (2016). Transcriptional reprogramming and phenotypic switching associated with the adaptation of *Lactobacillus plantarum* C2 to plant niches. Sci Rep.

[105] Kahala M, Ahola V, Mäkimattila E (2014). The use of macroarray as a simple tool to follow the metabolic profile of *Lactobacillus plantarum* during fermentation. Adv Microbiol.

[106] Riveros-Mckay F, Campos I, Giles-Gómez M (2014). Draft genome sequence of *Leuconostoc mesenteroides* P45 isolated from Pulque, a traditional mexican alcoholic fermented beverage. Genom Announ.

[107] Giles-Gómez M, García JGS, Matus V (2016). In vitro and in vivo probiotic assessment of *Leuconostoc mesenteroides*. SpringerPlus.

[108] Chiou TY, Oshima K, Suda W (2016). Draft genome sequence of *Lactobacillus farciminis* NBRC 111452, isolated from *kôso*, a Japanese sugar-vegetable fermented beverage. Genom Announ.

[109] Chiou TY, Suda W, Oshima K (2016). Changes in the bacterial community in the fermentation process of *kôso*, a Japanese sugar-vegetable fermented beverage. Biosci Biotechnol Biochem.

[110] Gram L, Ravn L, Rasch M (2002). Food spoilage—interactions between food spoilage bacteria. Int J Food Microbiol.

[111] Temitope FP, Oluchi UE (2015). Studies of the antifungal activity of *Lactobacillus plantarum* and *Lactobacillus fermentum* on spoilage fungi of tomato fruit. J Microbiol Res.

[112] Crowley S, Majony J, van Sinderen D (2013). Broad-spectrum antifungal-producing lactic acid bacteria and their application in fruit models. Folia Microbiol.

